# A novel field method for preserving African lion (*Panthera leo*) fecal samples for noninvasive hormone metabolite analysis^,^^✰^^✰✰^

**DOI:** 10.1016/j.mex.2022.101881

**Published:** 2022-11-01

**Authors:** Katherine J. Fowler, Rachel M. Santymire

**Affiliations:** a Department of Biological Sciences, University of Illinois at Chicago, 845 W. Taylor Street, Chicago, IL 60607, United States; b Biology Department, Georgia State University, Atlanta, GA, United States

**Keywords:** Endocrinology, Carnivore, Reproduction, Glucocorticoids, Validation, Desiccation, Silica

## Abstract

The traditional methods of preserving fecal samples to suspend hormone-degrading bacteria are not always options during remote fieldwork when studying wildlife endocrinology. Our goal was to develop a field method for preserving African lion (*Panthera leo*) feces for hormone metabolite analysis by determining the stability of fecal hormone metabolites: 1) when exposed to the natural environment, and 2) in silica beads at ambient temperatures. We collected fresh feces from zoo-housed lions and combined them into two (male and female) homogenous samples. Each was divided into eight samples to undergo a cross-designed treatment.

• We immediately lyophilized one sample to serve as the control.
• We then exposed seven samples outside to natural environmental conditions for 0, 12, 24, 36, 48, 60, or 72 h. After outdoor exposure, we desiccated a subsample in silica beads for an additional 5, 7, or 10 days.
• We analyzed the fecal hormone metabolite concentrations in each sample using enzyme immunoassays for corticosterone, cortisol, testosterone, progesterone, and estradiol.

We determined that male and female fecal hormone metabolites in fresh African lion fecal samples are stable and comparable to a standardized desiccation method if dried in silica beads for 5 to 10 days prior to storing them at -20℃.

## Graphical abstract

**Figure:**
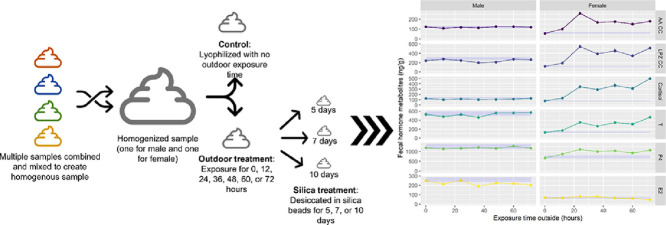

**Specifications table**

**Table:** 

Subject area	Agricultural and Biological Sciences
More specific subject area	*Zoology*
Name of your method	*Drying of fecal samples in silica beads prior to steroid hormone extraction and quantification.*
Name and reference of original method	*N/A*
Resource availability	Teaspoons: https://www.amazon.com/gp/product/B07J55SMQ2/ref=ppx_yo_dt_b_asin_title_o00_ s00?ie=UTF8&psc=1 Desiccation jars: https://www.amazon.com/gp/product/B07QS4K8MT/ref=ppx_yo_dt_b_asin_title_o01_ s00?ie=UTF8&psc=1 Mesh bags: https://www.amazon.com/gp/product/B073J4RS9C/ref=ppx_yo_dt_b_asin_title_o04_ s00?ie=UTF8&psc=1 Silica beads: https://www.sigmaaldrich.com/US/en/product/sial/s7500

## Method details

### Fecal sample collection

Animal care staff collected fresh fecal samples from one adult male lion (studbook number 383; 10.4 years old at beginning of study) and two adult female lions (studbook numbers 518 and 519; 6.8 years old at beginning of study) living at the Lincoln Park Zoo in Chicago, IL, USA. The care staff used gloved hands to put the samples into labeled, press-seal plastic bags that were immediately frozen at −20 °C until we had enough number of samples to create the male and female pooled samples. This sample collection required single-use plastic bags, which would not be used in the field method.

### Fecal sample processing

**Fig. 1:**
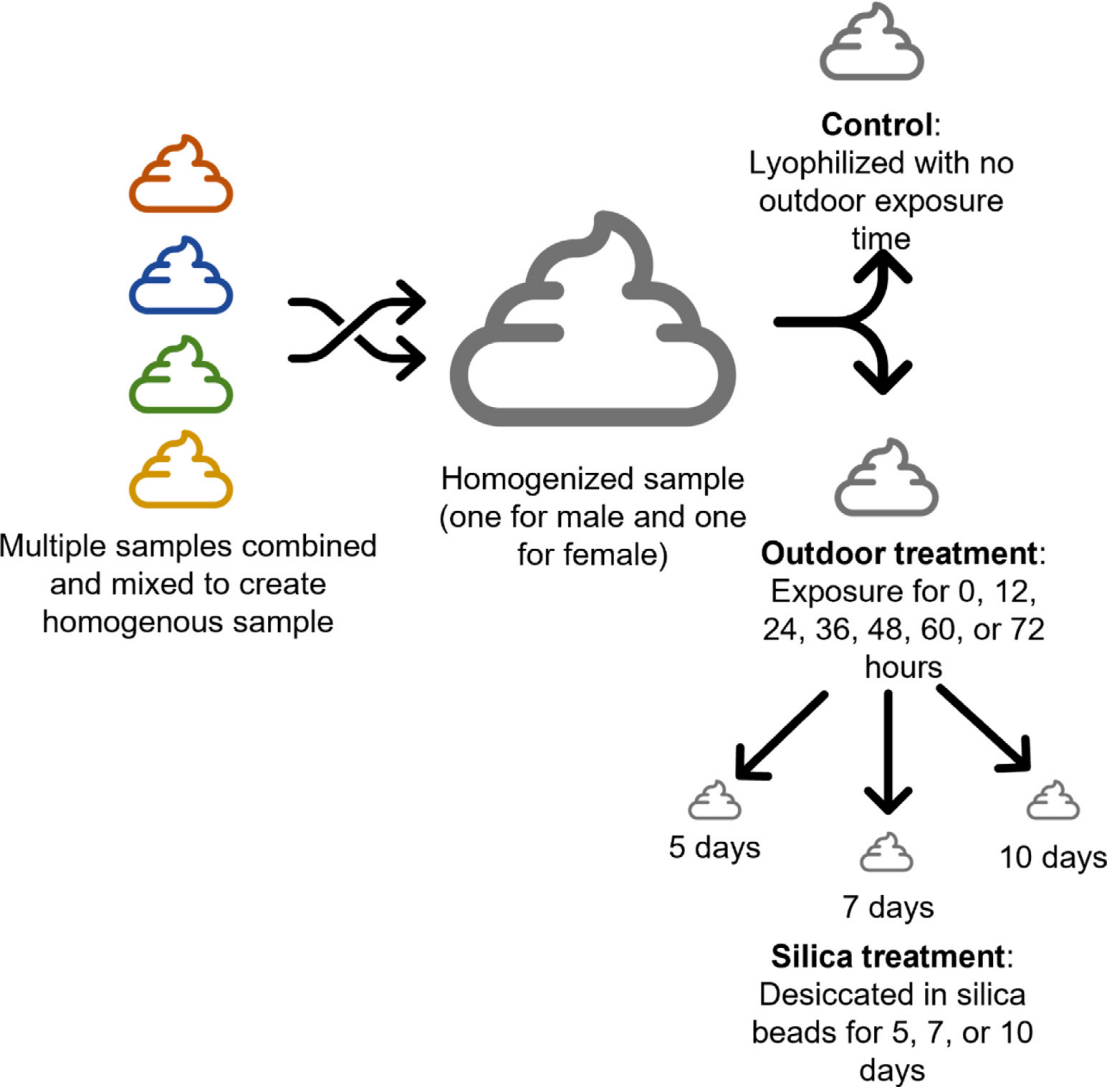
Lion fecal samples from one males and two female (Lincoln Park Zoo, Chicago, IL, USA) were mixed and homogenized into one large sample for each sex. Then the large samples were subdivided into the control sample that was immediately lyophilized and not exposed outside and samples for the outdoor treatment and then each outdoor treatment was subdivided into desiccation treatment in silica beads.

We thawed and mixed these samples into one large, homogenized pool each for male and female that we could then subset for the treatments. Each pool was then subdivided into treatments for the lyophilized control (0 h outside) or experimental samples exposed outdoors (0, 12, 24, 36, 48, 60, or 72 h) and then each sample per exposure time was desiccated in silica beads (5, 7, or 10 days) (Fig. 1). We developed an equation to determine how much wet feces we needed to start with to end with enough dry feces for the hormone metabolite quantification using Eq. (1).

(1) $$W=\frac{M*D}{1-L}$$

Here, *M* is the number of multiples of the extract needed, *D* is the weight of dry feces required per the extraction protocol, *W* is the weight of wet feces to be put in silica beads, and *L* is the proportion of water mass lost during desiccation. For our study, we wanted 10 times the amount needed for extraction, our extraction protocol uses 0.2 g dry feces, and we determined desiccation through lyophilization resulted in a 60% loss in mass, meaning we needed to start with 5 g of wet feces for each silica bead treatment.

**Fig. 2:**
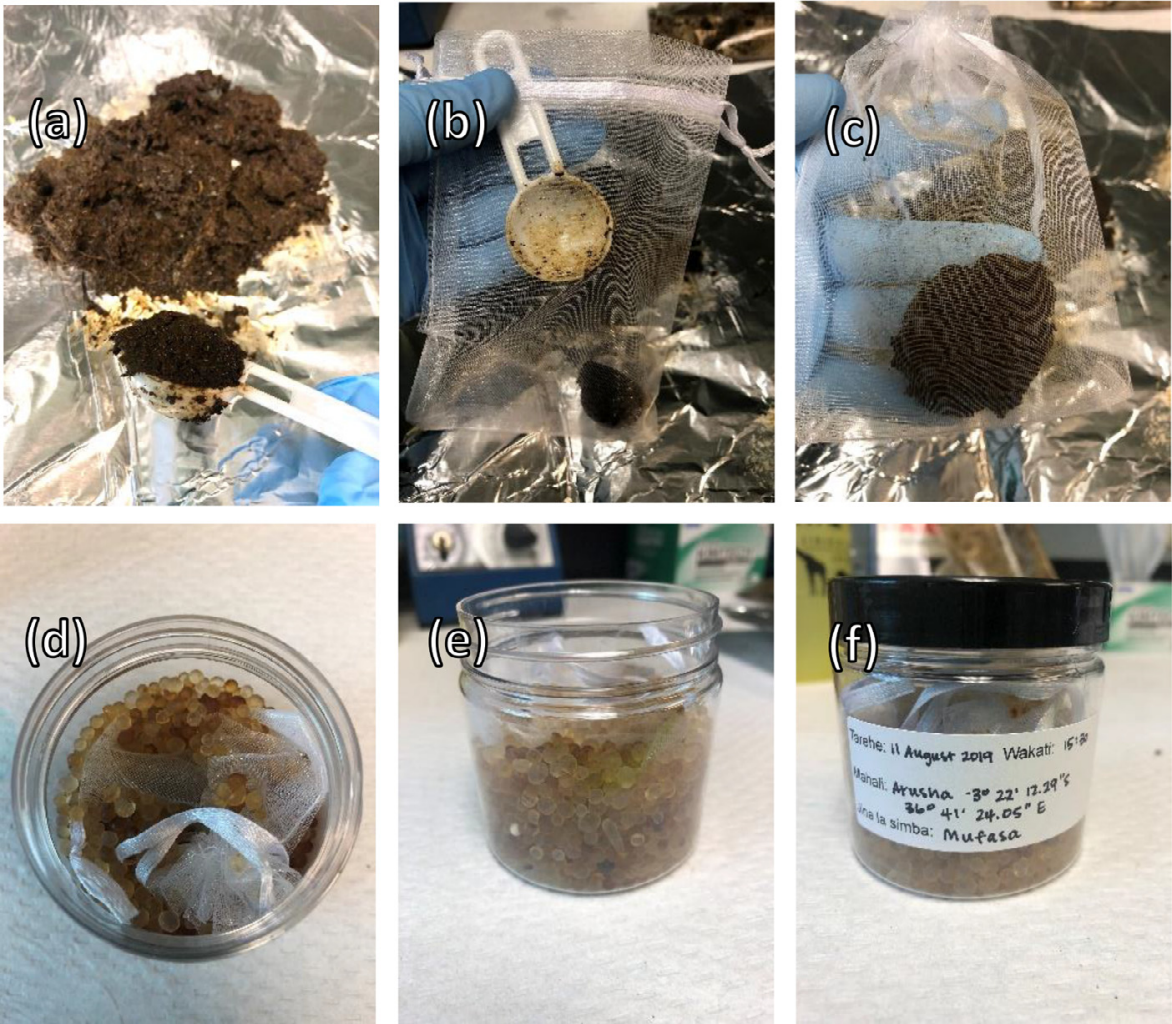
Steps for collecting and processing an African lion fecal sample using this field method: (a) mix entire sample on foil and take one teaspoon of sample, (b) put one teaspoon of sample into mesh organza drawstring bag, (c) close drawstring and gently press feces flat to increase surface area, (d) put mesh bag with sample into plastic jar with silica beads, (e) ensure sample bag is buried well and surrounded by silica beads, (f) close jar and label with relevant sample information.

The large, pooled sample was reformed into smaller samples made to the average size of a piece of lion feces from the zoo lions (5.5 cm x 3 cm x 3.5 cm, with < 1 cm total difference) to best replicate a natural defecation scenario. To reproduce the time between when a lion defecates until it is collected by the field team, these samples were exposed to outdoor summer conditions in Chicago, IL, USA which experiences comparable weather to the study area in Tanzania, for 0, 12, 24, 36, 48, or 72 h. During the dates that samples were exposed, temperatures ranged from 18 to 23 °C and there was a total of 2.2 cm of rain (www.weather.gov). We acknowledge that temperatures in lion native ranges can be both higher and lower than the temperatures during our study. We encourage future studies to include more extreme temperatures if that better suits the field environment. We created a control sample that was not exposed outdoors and was desiccated using a lyophilizer (ModulyoD, Thermo Fisher Scientific, Inc., Waltham, MA), which is a previously validated method for lion fecal hormone metabolite extraction [1]. For the experimental samples, after the sample spent its specified time outdoors, we processed the sample as it would be in the field using Hunt & Wasser (2003) as a guide while incorporating their suggestions and our requirements and limitations. We mixed the sample on aluminum foil and put one teaspoon weighing approximately 5 g into a mesh organza drawstring bag (10.1 × 15.2 cm, Amazon.com, USA), flattened it to increase the surface area, and submerged it in 25 g silica beads (3.5 mm, Type II, silica gel, Sigma Aldrich Chemical #S7500, St. Louis, MO, USA) in clear, reusable plastic screw-top jar (6.5 cm x 6.5 cm, 118 mL, Amazon.com, USA) to create a feces to silica beads ratio of 1:5 for either 5, 7, or 10 days at room temperature and then stored it at −20 °C until analysis (Fig. 2). fter the samples were desiccated, they were all treated the same. The dried samples were crushed in plastic 16×100 mm test tubes using glass rods. Any bones or non-fecal debris were removed. We extracted the samples using established methods [1,3]. In short, 0.2 ± 0.02 g of dry feces is extracted twice with 5 mL of 90% ethanol:distilled water, centrifuged, and the combined supernatants were dried down and reconstituted in 1 mL of methanol and further diluted as needed using phosphate buffered saline (0.2 M NaH_2_PO_4,_ 0.2 M Na_2_HPO_4_, NaCl).

### Hormone metabolite analysis

We quantified the fecal hormone metabolites using enzyme immunoassays (EIAs) for 5 hormones using 6 assays. We measured fecal glucocorticoid metabolites using corticosterone (CC) using an in-house (LPZ CC) and commercial assay (AA CC) and cortisol (CORT), fecal androgen metabolites using testosterone (T), fecal progestogen metabolites using progesterone (P4), and fecal estrogen metabolites using estadriol-17β (E2; Table 1). We validated the EIAs by demonstrating: 1) parallelism between binding inhibition curves of fecal extract dilutions, and 2) significant recovery (> 90%) of exogenous hormone added to fecal extracts.

**Table 1:** The enzyme immunoassays used to quantify fecal hormone metabolites in African lion feces during the field method validation study. Arbor Assays, Inc. is in Ann Arbor, MI, USA. Reagents from Coralie Munro were made at University of California, Davis, CA, USA.

Hormone	Antiserum ID	Antiserum & conjugate source	Antiserum concentration	HRP concentration	See for cross-reactivities	Sensitivity
Corticosterone	polyclonal, C044	Arbor Assays, Kit K014	use per kit protocol	use per kit protocol	Arbor Assay K014 kit protocol	39.06 pg/mL
Corticosterone	polyclonal, CJM006	Coralie Munro	1:225,000	1:200,000	Santymire and Armstrong 2010	3.9 pg/50uL
Cortisol	polyclonal, R4866	Coralie Munro	1:375,000	1:200,000	Loeding et al. 2011, Young et al. 2004	1.95 pg/50uL
Testosterone	polyclonal, R156/7	Coralie Munro	1:750,000	1:375,000	Loeding et al. 2011	0.85 pg/50uL
Progesterone	monoclonal, CL425	Coralie Munro	1:70,000	1:750,000	Loeding et al. 2011	0.78 pg/50uL
Estradiol-17β	polyclonal, R0008	Coralie Munro	1:250,000	1:200,000	Amaral et al. 2013	1.95 pg/20uL

### Data analysis

The statistical analyses were performed using *R Studio* (version 3.5.1) with a significant value of 0.05. All hormone metabolite concentrations are reported as mean ± standard error in nanogram of hormone metabolite per gram of dry feces (ng/g). We used a randomized block ANOVA to determine the effects of exposure time and silica bead treatments. Because we were not able to do replicates for each treatment, if there was no difference (*P* > 0.05) in any of the silica beads treatments, the samples would be considered replicates and the mean would be used to determine comparability to the control. To determine if the samples desiccated in silica beads were comparable to our lyophilized control, we decided that the final concentration in ng/g must be within ± 10% of the lyophilized hormone metabolite concentration since our laboratory used a 10% difference allowed variability in other validations.

## Method validation

### Laboratory biochemical validations

All six EIAs passed our validation requirements (Table 2).

**Table 2:** Parallelism and percent recovery validation results from the six enzyme immunoassays in this study.

Hormone assay	Parallelism^a^	Percent recovery^b^
Corticosterone (AA CC)	0.990	*y* = 0.94x + 10.069; R^2^ = 0.9972
Corticosterone (LPZ CC)	0.944	*y* = 0.86x + 1.6434; R^2^ = 0.9953
Cortisol (CORT)	0.999	*y* = 1.03x - 5.67; R^2^ = 0.9992
Testosterone (T)	0.981	*y* = 1.09x - 7.10; R^2^ = 0.9880
Progesterone (P4)	0.982	*y* = 0.88x - 4.75; R^2^ = 0.9915
Estradiol-17β (E2)	0.997	*y* = 1.00x - 7.85; R^2^ = 0.9877

### Silica beads treatment

For male and female samples, hormone metabolite concentrations were similar (*P* > 0.05) across all samples for time spent in silica beads (Table 3). We did not note any mold growth on the treatment samples during the study. When the samples were finished desiccating, they felt dry and were easy to crush with the glass rod regardless of having spent 5, 7 or 10 days in the silica beads. Some samples stuck to the mesh organza bag but could be removed without damaging the bag.

**Table 3:** Statistical results comparing enzyme immunoassay results using randomized-block ANOVA blocked by outdoor exposure time and time desiccated in silica beads; control range is ± 10% of lyophilized control concentrations and treated sample range is for samples exposed outside and desiccated in silica beads.

Hormone, sex	Exposure time block, F value	Exposure time block, P value	Silica beads time block, F value	Silica beads time block, P value	Control range (ng/g)	Treated sample range (ng/g)
AA Corticosterone, male	0.936	0.504	1.791	0.209	107.3 - 131.1	86.1 - 135.2
Corticosterone, male	0.849	0.557	2.205	0.153	258.6 - 316.1	197.5 - 302.8
Cortisol, male	1.284	0.334	0.166	0.849	120.5 - 147.3	93.6 - 151.6
Testosterone, male	1.298	0.329	3.359	0.069	522.3 - 638.4	370.7 - 775.6
Progesterone, male	2.625	0.073	1.938	0.187	1128.7 - 1379.5	1079.5 - 1345.4
Estradiol-17β, male	1.969	0.15	1.204	0.334	235.7 - 288.0	165.8 - 296.8
AA Corticosterone, female	10.627	< 0.001	0.367	0.700	53.9 - 65.8	52.2 - 284.0
Corticosterone, female	25.836	< 0.001	1.482	0.266	101.8 - 124.4	94.2 - 608.9
Cortisol, female	36.168	< 0.001	1.737	0.218	65.9 - 80.6	62.5 - 519.2
Testosterone, female	59.19	< 0.001	0.77	0.485	119.9 - 146.5	120.0 - 528.6
Progesterone, female	21.681	< 0.001	0.047	0.954	662.9 - 810.3	600.9 - 1166.5
Estradiol-17β, female	3.271	0.0382	0.098	0.907	60.7 - 74.2	40.7 - 92.4

### Outdoor exposure time

**Fig. 3:**
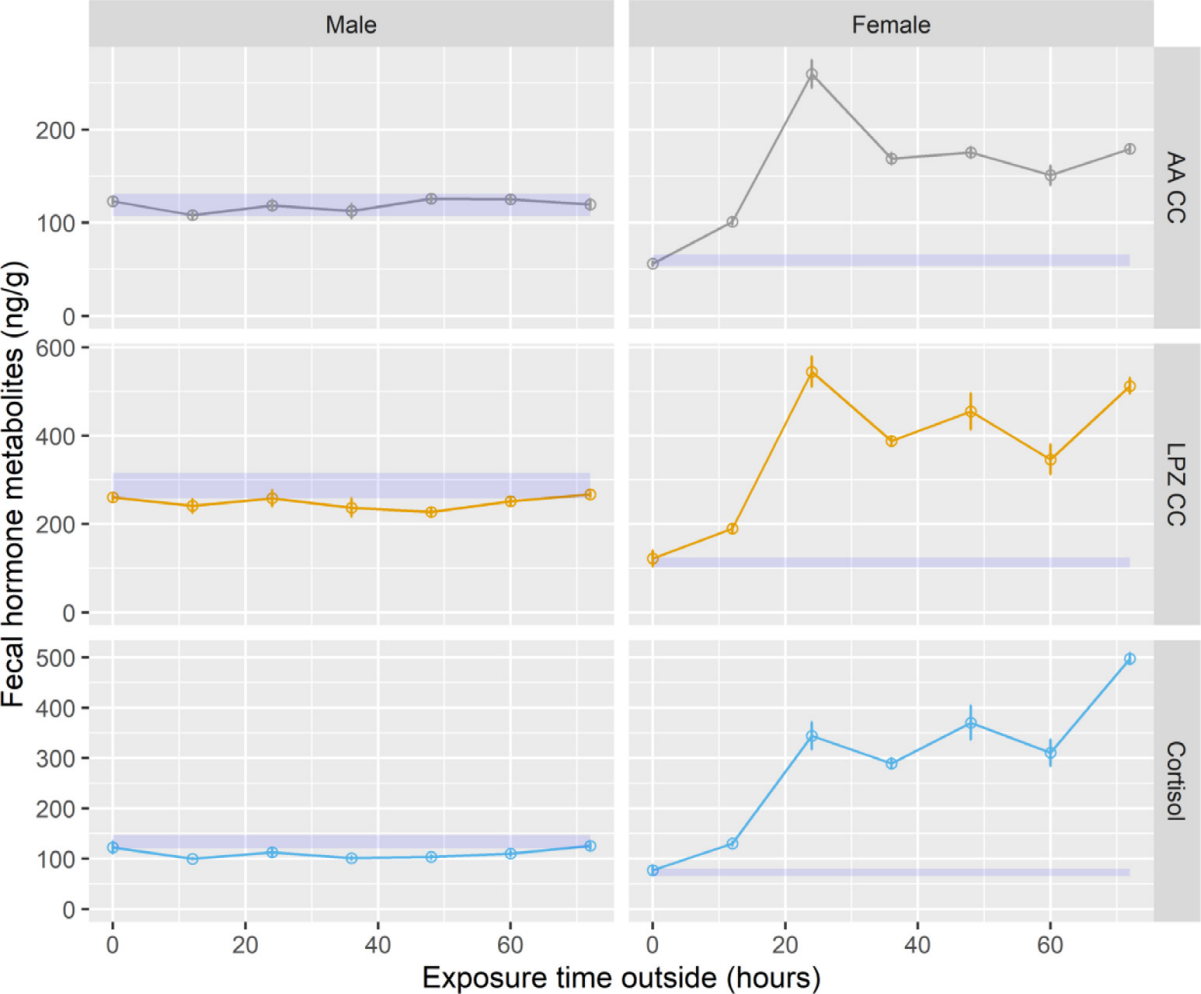
Means (±SE) of fecal hormone metabolites for African lion fecal samples exposed to outside conditions (0, 12, 24, 36, 48, 60, 72 h) and desiccated in silica beads (5, 7, 10 days) for three enzyme immunoassays measuring glucocorticoids: Arbor Assays corticosterone (AA CC), in-house corticosterone (LPZ CC), and in-house cortisol (Cortisol). The shaded blue area indicates the acceptance range using the concentration of the lyophilized control sample ± 10%.

**Fig. 4:**
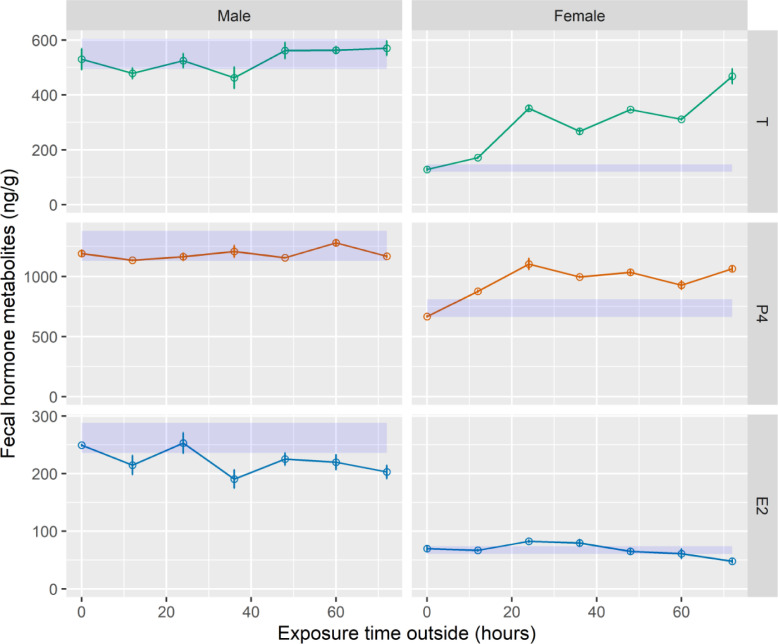
Means (±SE) of fecal hormone metabolites for African lion fecal samples exposed to outside conditions (0, 12, 24, 36, 48, 60, 72 h) and desiccated in silica beads (5, 7, 10 days) for three enzyme immunoassays measuring reproductive hormones: in-house testosterone (T), in-house progesterone (P4), and in-house 17β-estradiol (E2). The shaded blue area indicates the acceptance range of the concentration of the lyophilized control sample ± 10%.

Because the three silica bead treatments were similar, we treated them as replicates for each outdoor exposure time treatment and used the mean at each exposure time to compare to the control. For the male samples, fecal hormone metabolite concentrations for all outdoor exposure times for all EIAs were the same (*P* > 0.05; Table 3). When we compared the male samples to the control, all exposure times at 0 h for all hormones fell within the control range, but some time points at 12 h and after were not within the range (LPZ CC: 12, 24, 36, 48, 60 h; CORT: 12, 36, 48 h; T: 12, 36 h; E2: 12, 36, 48, 60, 72 h; Fig. 3, Fig. 4) because the hormone metabolite concentrations were too low.

For the female samples, fecal hormone metabolite concentrations increased (*P* < 0.05) with longer exposure time for all hormones, except E2 (Table 3). For the E2 EIA, outdoor exposure times were the same (*P* = 0.123). Compared to the control, all samples at time point 0 h were within the acceptable range, but most samples were not acceptable after 12 h of outdoors exposure (AA CC: 12, 24, 36, 48, 60, 72 h; LPZ CC: 12, 24, 36, 48, 60, 72 h; Cortisol: 12, 24, 36, 48, 60, 72 h; T: 12, 24, 36, 48, 60, 72 h; P4: 12, 24, 36, 48, 60, 72 h; E2: 24, 36, 72 h; Fig. 3, Fig. 4) mostly because they were much higher than the control range. For AA CC, LPZ CC, P4 and E2, hormone metabolite concentrations peaked at 24 h of exposure and then decreased from 36 to 72 h of exposure. For CORT and T, hormone metabolite concentrations were highest after 72 h of exposure. The peaks were approximately 1.5 to 7-fold higher than the 0 h exposure, excluding E2.

We determined that fresh African lion fecal samples can desiccated in silica beads for 5 to 10 days prior to storing them at −20℃ and the extracted fecal hormone metabolites are comparable to a standardized desiccation method using a lyophilizer. This preservation technique is useful for field work where there is no immediate access to a freezer, nor can the sample be extracted the day it is collected, and the collection and processing equipment must be small and mobile.

### Additional information

Hunt and Wasser [2] attempted using silica beads as a desiccation method prior to analyzing fecal hormone metabolites for African elephant (*Loxodonta africana*) and grizzly bear (*Ursus arctos horribilis*) but only the elephant samples were comparable to the control. Perhaps, this related to the varying diets, which can affect the water content of the fecal material. We considered their methods and implemented some changes, such as increasing the wet feces to silica beads ratio from 1:3 and 1:4 to 1:5 to encourage faster desiccation and prevent mold growth. We also used mesh organza bags to contain the wet feces instead of coffee filter paper to attempt to increase drying action of the silica and decrease the possibility of any material sticking to the samples. We also stored our samples at −20 °C after they were desiccated.

This validation is an important reminder how fecal hormone metabolites can vary between individuals and sexes [4]. Regarding the silica beads treatments, male and females samples showed no difference between desiccation in silica beads for 5, 7, or 10 days and were dry by the 5th day. When the samples were exposed to the outside elements, for both the male and female samples, only at 0 h of outdoor exposure were the samples within the acceptable range of the lyophilized control for all hormones tested which demands that samples be collected fresh. For the male, some of the samples with longer outdoor exposure times fell within the acceptable range and more of the samples were closer to the range than the female samples. The male samples were either within the range or below it. It is possible with more replicates of the lyophilized control and the treatment samples that more of the male samples would have been within the acceptable range. For the female samples, except for E2, all samples outside of the acceptable range had much higher concentrations. These sex differences in hormone metabolite stability could be attributed to differences in water intake with possibly the male samples being less hydrous. Additionally, there have been noted sex differences in fecal glucocorticoid metabolite production in laboratory mice (*Mus musculus f. domesticus*) [5], while no sex differences were found in jaguar (*Panthera onca*) [6].  However, the fact that exposure times at and after 12 h were no longer comparable to the control is consistent with previous validations in other species using other techniques. Stetz et al. [7]found that in free-ranging grizzly bears and black bears (*Ursus americanus*), fecal glucocorticoid metabolites decreased with exposure to precipitation and increased with exposure outdoors, even though Kinoshita [8] found that ultraviolet exposure did not influence hormone metabolite concentrations in snow leopard feces. For African elephants, fecal glucocorticoid metabolites decreased, and fecal progestogen and estrogen metabolites increased as time since defecation increased [9]. Similarly for brown hyena (*Hyaena brunnea*), fecal glucocorticoid metabolites decreased as time since defecation increased [10]. For the silica bead treatments, all the male and female samples were the same regardless of spending 5, 7, or 10 days at room temperature in silica beads. This was a positive result since different methods of desiccation were not comparable in cheetah feces [11]. Our results were in line with other studies of mammalian fecal hormone studies that outdoor exposure destabilized fecal hormone metabolite concentrations which is not surprising, but still worth testing since time and flexibility are valuable resources during field work.

Previous research on African lion hormone production also used fecal samples collected as fresh as possible for free-ranging lions [12] and samples <24 h old for captive lions [13,14]. Creel et al. [12] used liquid nitrogen to preserve African lion fecal samples and found that male lions had higher fecal glucocorticoid metabolite concentration than females when cubs were absent, which is also true regarding our hormone metabolite results for both corticosterone EIAs and the cortisol EIA and there were no cubs in this pride when we collected the samples for our study. While the results by Putman et al. [13,14] are interesting, their study designs do not allow us to make hormone concentrations comparisons.

### Recommendations

There are several action items we recommend to people planning to replicate this study. The initial titration is important for determining the ratio of wet feces to silica beads to control the desiccation method so that the feces dry quickly; otherwise, the sample will start to mold and not usable for hormone metabolite extraction. For materials selection, if a study is being conducted in an area where trash is burned, use wax or parchment paper instead of foil since it will burn more easily. Additionally, choose a screw-top container that minimizes the headspace between silica beads and top of the jar. Similarly, choose the smallest mesh organza bag that can still fit your sample, so it is easiest to bury in the silica beads. For experimental set-up, when the samples are exposed outside, try including sun, shade, and precipitation as measurable variables in the study. Since we determined that fresh samples must be used for our study, these variables were negated, but exploring all conditions is a worthwhile endeavor. Also, perform multiple replicates on the control and for each treatment. We also recommend planning smaller time increments, especially between the 0 and 12 h of exposure outdoors. It would be useful if the collector observes an animal defecate, can note the sample location, and then return some hours later when the animal has moved away and collect the sample. We also encourage scientists to plan field work protocols in a way that local people of all scientific skill levels can be hired to work on their projects. This includes considering how to reduce language barriers, designing methods that can completed by people with different training backgrounds, and sourcing supplies from local vendors.

## Ethics statements

All work was conducted in accordance with NIH and the Office of Laboratory Animal Welfare under Assurance Number D16–00290 (A3460.01) under Animal Care Committee protocol number 21–152.

## Credit author statement

***Katherine Fowler*** *: Conceptualization, Methodology, Validation, Formal analysis, Investigation, Resources, Writing – Original Draft, Visualization, Funding acquisition.* ***Rachel Santymire*** *: Writing – Review & Editing, Supervision.*

## Funding

This work was supported by the University of Illinois Provost's Graduate Research Award and the Elmer Hadley Graduate Research Award.

## Declaration of Competing Interest

The authors declare that they have no known competing financial interests or personal relationships that could have appeared to influence the work reported in this paper.

## Data Availability

Data will be made available on request.
